# Highly Thermally Conductive Triple-Level Ordered CNT/PVA Nanofibrous Films

**DOI:** 10.3390/polym16060734

**Published:** 2024-03-07

**Authors:** Yanyan Wu, Anqi Chen, Wenlong Jiang, Zhiye Tan, Tingting Fu, Tingting Xie, Guimei Zhu, Yuan Zhu

**Affiliations:** 1 School of Microelectronics, Southern University of Science and Technology, Shenzhen 518055, China; 2 School of Innovation and Entrepreneurship, Southern University of Science and Technology, Shenzhen 518055, China; 3 Academy for Advanced Interdisciplinary Studies, Southern University of Science and Technology, Shenzhen 518055, China

**Keywords:** thermal management, poly(vinyl alcohol)-based materials, Angstrom method

## Abstract

The escalating thermal power density in electronic devices necessitates advanced thermal management technologies. Polymer-based materials, prized for their electrical insulation, flexibility, light weight, and strength, are extensively used in this field. However, the inherent low thermal conductivity of polymers requires enhancement for effective heat dissipation. This work proposes a novel paradigm, emphasizing ordered structures with functional units, to create triple-level, ordered, low-filler loading of multi-walled carbon nanotube (MWCNT)/poly(vinyl alcohol)(PVA) nanofibrous films. By addressing interfacial thermal resistance through –OH groups, the coupling between polymer and MWCNT is strengthened. The triple-level ordered structure comprises aligned PVA chains, aligned MWCNTs, and aligned MWCNT/PVA composite fibers. Focusing on the filler’s impact on thermal conductivity and chain orientation, the thermal transport mechanisms have been elucidated level by level. Our MWCNT/PVA composite, with lower filler loadings (10 wt.%), achieves a remarkable TC exceeding 35.4 W/(m·K), surpassing other PVA composites with filler loading below 50 wt.%.

## 1. Introduction

**Figure 1:**
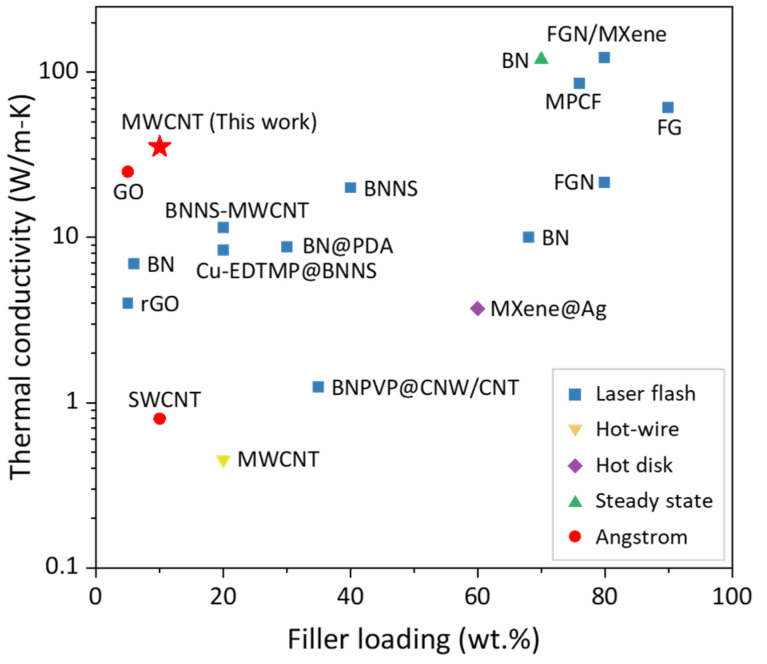
Comparison of the in-plane TC of PVA composite with different fillers.

The continual increase in the thermal power density of electronic devices poses a great challenge to thermal management technology [1,2]. Polymer-based thermal management materials, with the advantages of a good electrical insulation performance, flexibility, light weight, and high strength, have attracted a lot of attention and are widely used in the thermal management field. However, the thermal conductivity (TC) of polymers is too low (~0.3 W/(m·K)) to meet the thermal conductive requirements [3,4,5]. Here are two pathways to improve it. One is to tune the aggregation state of polymer materials to provide an efficient phonon transport path to enhance TC [6,7]. Another method is to add high-thermal-conductivity fillers into the polymers. The fillers are usually mixtures of ceramic powders [8,9,10], metal powders [11,12,13], or even diamond powders [14,15]. Practically, the TC of polyethylene (PE, [–CH_2_–CH_2_–]_n_) fibers and films can achieve 104 W/(m·K) [16] and 62 W/(m·K) [17], respectively, which is the highest among the pure polymers because of its simple C–C bond. However, PE is not suitable for practical thermal management applications due to its low melting temperature (~120 °C), where the aging condition of most application scenarios reaches 150~180 °C. In our previous work [18], poly(vinyl alcohol) (PVA, [–CH_2_–CHOH–]_n_) with a melting temperature higher than 200 °C was introduced. The bulk TC was enhanced from ~0.3 W/(m·K) to 8.51 W/(m·K) because it preserves the carbon chain as an efficient phonon transport path and introduces only minor changes (e.g., –OH in this work) [19,20]. Furthermore, a number of low-dimensional materials were composited with PVA for a thermal conductivity investigation, including carbon nanotube (CNT) [21,22], carbon fiber [23], boron nitride (BN) [24,25,26,27,28], MXene [29], fluorographene nanosheets (FGN) [30,31], graphite oxide (GO) [32,33], and their composites [34,35,36,37]. The composite materials show high TCs ranging from 1 to more than 100 W/(m·K) at different loadings (shown in Figure 1). BN, FG, and MXene can reach high loadings for more than 70 wt.%. The FGN/MXene/PVA [34] composite with the 80 wt.% filler content achieves the highest TC of 122.5 W/(m·K). However, the high filler loading incurs aging risks such as dryout or cracking. Thereby the low-loading composites, although they may have a lower TC, are more pragmatic choices. In PVA composites with loadings of less than 50 wt.%, multi-walled CNT filler is one of the most popular TC enhancements. However, it is not as high as expected, especially when the TC of CNT theoretically reaches the range of 2000–6000 W/(m·K) [38]. It may be that the random arrangement and spontaneous clustering of CNTs make the CNT/polymer composites less thermally conductive [39,40]. 

**Figure 2:**
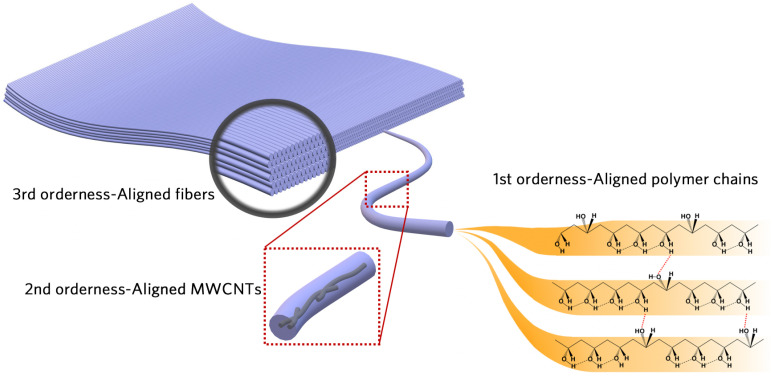
Schematic of triple-level orderness strategy for MWCNT/PVA composite fibrous film. The film is comprised of MWCNT@ PVA core-shell nanofibers. The 1st-level orderness is to align polymer chains; the 2nd-level orderness is to align the MWCNTs; and the 3rd-level orderness is to align fibers.

It is noteworthy that the aforementioned thermal conductivities are measured by different methods. We specify this information in Figure 1 using different markers. These works only pay attention to the loading and arrangement of fillers, which are two major strategies to enhance composite thermal conductivity. However, there is emerging research interest in polymers with a metal-like thermal conductivity, meaning that the polymer itself could be the crux of thermal enhancement. Here, we suggest a new paradigm, a triple-level ordered structure, to prepare low-filler-loading MWCNT/PVA nanofibrous films. The triple levels are shown in Figure 2: aligned PVA chains (1st-level), aligned MWCNTs (2nd-level), and aligned MWCNT/PVA composite fibers (3rd-level). The –OH group in PVA helps to couple the polymer and the MWCNT surface to reduce the interfacial thermal resistance. In previous work, aligned pure PVA chains (1st-level) were achieved using a three-step method and the TC could rise from 0.3 to 8.51 W/(m·K). Here, we provide more characteristic details of the first-level, and move forward to the second-level and third-level, orderness. On the 2nd-level, we pay special attention to the filler’s influence on the TC and chain orientation. The thermal transport mechanisms for the triple-level ordered composite are expounded level by level. The TC of our MWCNT/PVA composite with lower filler loadings (10 wt.%) reaches more than 35.4 W/(m·K), which is the highest among the PVA composites with a filler loading of less than 50 wt.%. This work shows promising progress in highly thermal conductive composites, which is a step forward to its true application.

## 2. Materials and Methods

### 2.1. Preparation of the MWCNT/PVA Composite Fibrous Films

Disentangling chains: The PVA (Mw ~145,000, *ρ* = 1.27 g/cm^3^, Aladdin) solid particles were dissolved into pure water under 4 h stirring at 60 °C. The as-obtained 10 wt.% solution (~25 mL) was then treated with an ultrasonic cell crusher (600 W, HDS-1000, Haoda, China) for half an hour. The ultrasonic process could break hydrogen bonds and drastically reduce the entanglement between PVA chains. After this treatment, the polymer chains are more prone to orientation along the electric field force during electrospinning.

Arranging MWCNTs: The MWCNTs (Chengdu Organic Chemistry Co., Ltd., CAS, Chengdu, China) used in this work have an average diameter of 10 ± 3 nm and length of 7 ± 2 μm, *ρ* = 1.62 g/cm^3^. A water-dispersing agent for carbon nanotubes with both an aromatic ring and hydrophilic group was used to improve the dispersibility. During the dispersing process, the aromatic ring can attach to MWCNTs, and the hydrophilic group improves the dispersibility of MWCNTs in water. Then, the proper amount of modified MWCNTs was added to the PVA solution and dispersed ultrasonically for half an hour to form solutions with different MWCNT/PVA concentrations (from 0 to 12 wt%). The volume fraction was then calculated using the weight fraction and the density of MWCNT and PVA.

Assembling fibers: Nanostructured film was produced by electrospinning. A pair of parallel electrode collectors was introduced to obtain highly orientated fibers. The needle was connected to the positive terminal of the first power generator, and the parallel electrodes, as the collection target, were connected to the negative terminal. The voltage applied for electrospinning is 12 kV and the distance between the electrodes is 12 cm.

Stretching films: the nanostructured composite films were mechanically stretched at ~200 °C using homemade uniaxial stretching equipment with an infrared heating chamber, and the stretching rate was about 1 cm/min.

### 2.2. Characterizations

The FTIR experiments were performed by Fourier transform infrared spectrometer (Nicolet IS50, Thermo Fisher Scientific, Waltham, MA, USA). The range of the light wavelength was 2.5 µm~25 µm. The light incident angle was 90° to the film. The morphology of samples was characterized by scanning electron microscope (SEM, ZEISS Merlin, Stuttgart, Germany). The arrangement of the MWCNTs was obtained by transmission electron microscope (TEM), (FEI, Tecnai G2 spirit 120 kV, Hillsboro, OR, USA). The viscosity was measured using viscometer NDJ-9S (Yueping, China), followed by GB/T 2794-2022 testing method. An online laser diffraction system was set up to monitor the orientation of the samples. The diameter of fibers is ~200 nm; thus, a blue laser (wavelength 405 nm) was used, and the power was set at 100 mW to avoid film damage. The laser was placed 10 cm away from the film and the laser beam (about 2 × 2 mm^2^) was nearly perpendicular incident to the film. A black screen was placed on the other side of the film to collect diffraction patterns. The alignment degree *f*, which is also called Herman’s Orientation Factor (HOF), can be calculated by the intensity distribution in the diffraction pattern [41]:

(1) $$f=\frac{3\langle {cos}^{2}\theta \rangle -1}{2}$$

(2) $$\langle {cos}^{2}\theta \rangle =\frac{\int_{0}^{\pi /2}I(\theta ){cos}^{2}\theta sin\theta d\theta }{\int_{0}^{\pi /2}I(\theta )sin\theta d\theta }$$

where θ is the angle between the structural unit vector and the reference direction, and I(θ) is the intensity profile of anisotropy as a function of θ from 0 to π/2 in the laser diffraction patterns or Fourier transform of SEM images. 

The thermal conductivities ($K={\rho C}_{\rho }\alpha$)K of PVA fibers were obtained by separately measuring the diffusivity α, density ρ, and heat capacity $C_{\rho }$. The specific heat capacity could be obtained by differential scanning calorimetry (DSC) (MDTC-EQ-M06-01). The density is investigated by the Archimedes method. The Angstrom method was used for α measurement. In the Angstrom method [42,43], one end of the sample is heated by a sine wave. When the temperature oscillations stabilize, measurements of the amplitude (*M* and *N*) and phase delay (*dt*) of the temperature oscillations at two different spots (at a distance of *L*) along the sample enable calculation of the thermal diffusivity of the sample.

(3) $$\alpha =\frac{L^{2}}{2dtln(M/N)}$$

## 3. Results and Discussion

### 3.1. First-Level Orderness (Aligned PVA Chains)

**Figure 3:**
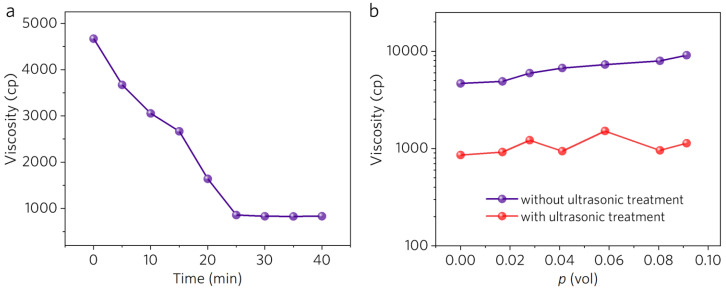
(**a**) The viscosity of PVA spinning solution as a function of treatment time. (**b**) The viscosity of MWCNT/PVA spinning solution as a function of MWCNTs volume fraction with (30 min) and without ultrasonic pretreatment.

In our previous work [18], we used high-power ultrasonic pretreatment to break the hydrogen bonds and disentangle the polymer chains. The polarized Fourier transform infrared (FTIR) and the wide-angle X-ray scattering (WAXS) results verified that ultrasonic pretreatment can significantly enhance the chain-aligning efficacy of electrospinning. Thus, here, ultrasonic pretreatment was also applied to the mixture of PVA solution and MWCNTs. However, FTIR and WAXS are posterior measurements. In practice, we need a real-time indicator of the disentanglement degree while processing. Viscosity measurement was found to be an effective means for this. Figure 3a shows the viscosity change in the pure PVA solution following different ultrasonic treatment times. With the increase in treatment time from 0 min to 25 min, the viscosity of the solution decreases significantly, from 4670 cP to 858 cP, and then reaches a plateau. The distinct decrease indicates that the PVA chains are well disentangled. When the filler is added (Figure 3b), a distinct decrease still appears over a wide range of MWCNT volume fractions. 

### 3.2. Second-Level Orderness (Aligned MWCNTs)

**Figure 4:**
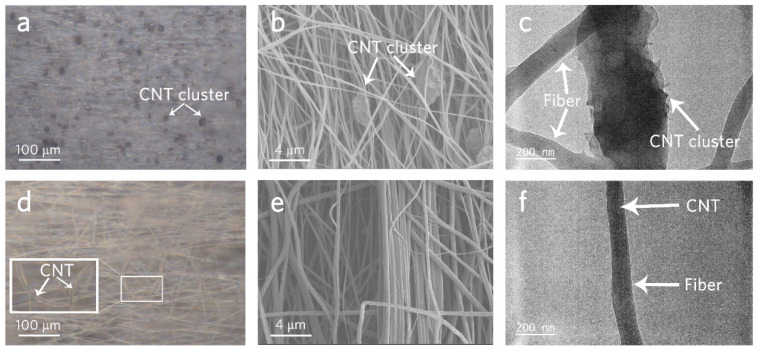
(**a**,**b**) Optical SEM images of unmodified MWCNT/PVA (*p* = 0.080) composite film with MWCNT clusters. (**c**) TEM image of the MWCNT cluster. (**d**,**e**) Optical SEM images of modified MWCNT/PVA (*p* = 0.080) composite film. (**f**) TEM image of the MWCNT@PVA core-shell fiber.

MWCNTs with a high surface energy are difficult to disperse and prone to aggregating or forming bundles [44]. In this work, chemical modification was used to help the MWCNTs’ dispersion process. In order to clarify the influence of MWCNTs on the TC, the features of MWCNT/PVA (*p* = 0.08) composite films with unmodified (Figure 4a–c) and chemically modified (Figure 4d–f) MWCNTs are compared in Figure 4. The unmodified MWCNTs aggregated, and the resultant clusters are clearly seen in optical microscope (Figure 4a), SEM (Figure 4b), and TEM (Figure 4c) images. In the TEM image, the cluster is composed of many coiled fibers. On the contrary, the modified MWCNTs disperse well in the polymer matrix and no black spot (cluster) could be found using the optical microscope (Figure 4d). Moreover, SEM images (Figure 4e) show that the modified MWCNT/PVA fibers are thicker than unmodified ones (Figure 4b), indicating that MWCNTs might be ‘in’ the fibers, which is observed and verified in the TEM image (Figure 4f). Figure 4f also shows that a MWCNT@ PVA core-shell fiber is formed. The TC of the composite fiber with aligned MWCNTs is 27.76 W/K-m, much higher than that of the fiber with aggregate MWCNT clusters (6.71 W/K-m).

**Figure 5:**
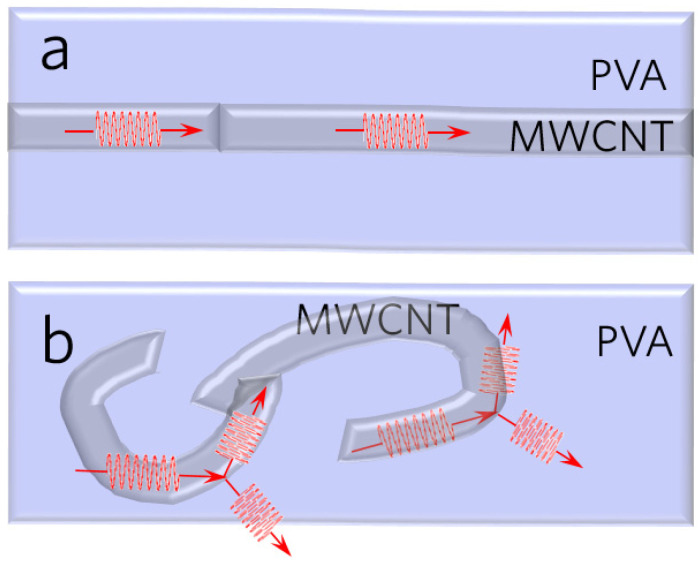
Phonon transport/scattering in the (**a**) MWCNT/PVA fiber with aligned MWCNTs; (**b**) MWCNT/PVA fiber with the MWCNT cluster composed of coiled MWCNTs.

This discrepancy in thermal conductivities is caused by phonon transport/scattering effects. MWCNT is the higher conductive phase in the composite; its TC is mainly composed of the longitudinal acoustic (LA) phonon [45,46]. Compared to the aligned MWCNTs in the fiber, more scattering in the coiled MWCNTs results in a decrease in the mean free path of the phonon (shown in Figure 5). Therefore, the axial TC of the composite film with MWCNT clusters comprised of a coiled MWCNTs is lower than that of the film with aligned MWCNTs. 

### 3.3. Inter-Level Perturbation

**Figure 6:**
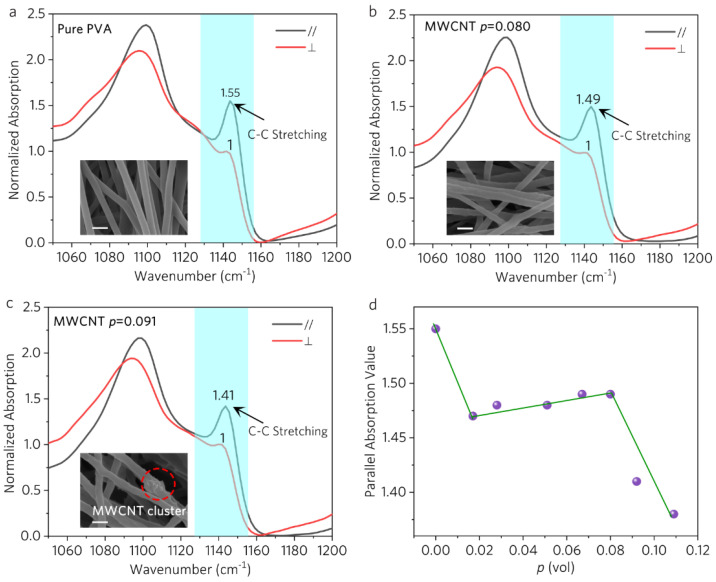
(**a**–**c**) FTIR spectra of the films with different modified MWCNT volume fractions (*p*) at two polarized IR configurations—parallel (//) and perpendicular (⊥). The parallel (//) configuration requires the IR polarization along the main axial direction of nanofibers. The perpendicular (⊥) configuration requires the IR polarization perpendicular to the axial direction. Insets: SEM images of the films and the scale bar is 300 nm. (**d**) The absorption values (1143 cm^−1^) as a function of volume fraction (*p*) at parallel (//) polarized IR configuration.

To understand the perturbation effect of MWCNT on the PVA chain alignment, polarized Fourier transform infrared (FTIR) measurement was used to semi-quantify the orientation degree of PVA chains. Figure 5 shows the FTIR spectra of PVA nanostructured film at two polarized IR configurations—parallel (//) and perpendicular (⊥). The C-C stretching band (1143 cm^−1^) [47] of the samples presents a distinct anisotropic feature: the perpendicular (⊥) polarized band intensity is much lower than the parallel polarizing (//) one. In Figure 6d, the degree of absorption anisotropy decreases and then stabilizes until *p* is larger than 0.080. This indicates that the addition of a proper amount (*p* < 0.080) of MWCNTs slightly breaks the alignment of the PVA chains, and the resulting TC loss is soon compensated by the gain from the MWCNTs themselves. Thus, there is no apparent TC drop in phase 2 (Figure 2). However, further addition of MWCNTs (*p* > 0.080) induces the rapid deterioration of the chain alignment; thus, in phase 3, an overdose of MWCNTs results in the sudden drop in TC. 

SEM images (insets of Figure 6a–c) show that the surface of the composite fibers is rougher compared to the pure PVA (inset of Figure 6a), implying that the MWCNTs weaken the PVA chain alignment. MWCNT clusters are only observed for *p* = 0.091 (inset of Figure 6c), which causes severe damage to the chain alignment and induces a sharp decrease in TC. In the solution, PVA chains can be treated as flowing past MWCNTs during the electrospinning process. For the dispersive MWCNTs, the single MWCNT tends to orient parallel to the flow direction due to the drag force of the flow, and laminar flow occurs in this case [48]. However, the large size of MWCNT clusters (approximately hundreds of nm) leads to the formation of turbulent wakes, which would significantly influence the alignment of PVA chains.

### 3.4. Third-Level Orderness (Aligned Nanofibers)

**Figure 7:**
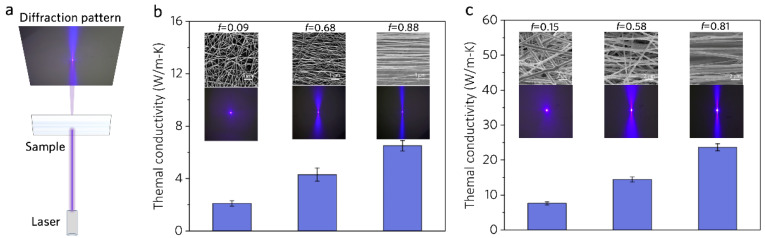
(**a**) Schematic of laser diffraction analysis system. SEM image, laser diffraction pattern, and TC of (**b**) pure PVA nanostructured film and (**c**) MWCNT/PVA (*p* = 0.080) nanostructured film with different alignment degrees.

The nanostructured film is composed of many nanofibers, so the nanofiber orientation factor is crucial to the TC. A laser diffraction analysis system [18] was set up to monitor the degree of nanofiber alignment online by observing the diffraction patterns (Figure 7a). In the diffraction pattern, the orientation information is mainly reflected in the azimuthal intensity distribution. The alignment degree *f*, which is also called Herman’s Orientation Factor (HOF), can be calculated using the intensity distribution in the diffraction pattern [44]. The value of *f* could be used to quantify the degree of alignment, where 0 and 1 represent random and perfect alignment, respectively. 

Figure 7b,c exhibit the TC of pure PVA and MWCNT/PVA (10 wt.%) nanostructured film with different *f* values. The highly aligned PVA nanostructured film with *f* = 0.88 has the highest TC (6.6 W/(m·K)), triple that of the randomly aligned nanostructured film (*f* = 0.09) (Figure 6b). Similar rules are observed for the composite MWCNT/PVA (*p* = 0.08) nanostructured film (Figure 6c). The highly aligned composite nanostructured film with *f* = 0.81 has a TC of 23.60 W/(m·K), almost quadruple that of the film with *f* = 0.15. These indicate that the TC of the film increases with the alignment degree of the fibers. Moreover, the random orientation and curvature of fibers may also lead to an increase in the phonon scattering at the fiber boundary, inducing a decrease in TC.

### 3.5. The Matrix of Triple-Level Orderness

**Figure 8:**
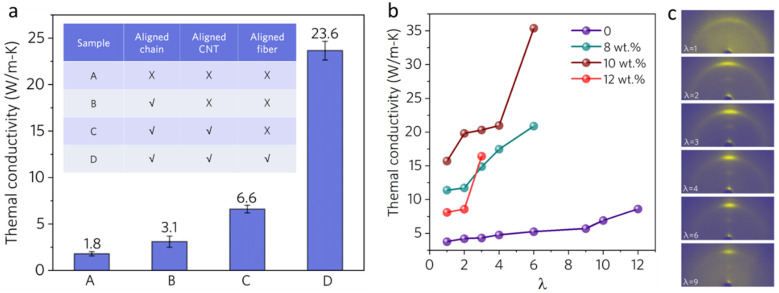
(**a**) TC of samples at different levels of orderness. (**b**) TC of samples with different MWCNT loadings as a function of draw ratio (λ). (**c**) WAXS patterns of composite with different draw ratios (λ).

Figure 8 shows the TC of the MWCNT/PVA (*p* = 0.080) nanostructured film with different levels of orderness. The TC of the MWCNT/PVA nanostructured film without any orderness is 1.8 W/(m·K). Along with the introduction of orderness to each level, the TC moves higher each step. The measured TC, as a function of the hot stretching ratio (λ), is depicted in Figure 8b. The TC of the nanostructured film increases further, from 15.7 W/(m·K) (λ = 1) to 35.4 W/(m·K) (λ = 6) (10 wt%), since hot stretching can improve the alignment of polymer chains and fibers, which has been shown in our previous works [18]. As shown in the WAXS pattern (Figure 8c), the relative intensity of the dispersion ring along the stretching direction diminished, while that perpendicular one gradually became concentrated, showing that the orientation of the composite increased with the increase in draw ratio. Compared to other polymer composites reported in Figure 1, our MWCNT/PVA composite exhibits the highest TC with a low loading.

## 4. Conclusions

In summary, we prepared high-thermal-conductive MWCNT/PVA composite films with triple-level orderness, including polymer chain level, MWCNT level, and fiber level. The best TC we achieved is 35.4 W/(m·K). The thermal transport mechanisms for the triple-level ordered composite were studied level by level. Furthermore, the inter-level perturbation, i.e., the perturbation effect of MWCNTs on PVA chains’ alignment, was specified. We found that the overdose of MWCNT (*p* > 0.080) can result in the deterioration or collapse of the PVA chain alignment, which causes a sharp drop in TC. The matrix of triple-level orderness verifies that each level plays an important role in the performance of composite materials and their full combination achieves the best performance. However, considering the inter-level perturbation, the processing parameters should be carefully chosen. This work provides a feasible way to enhance the thermal performance of the polymer-based composite, which is a step forward toward its true application in thermal management. 

## Data Availability

Data are contained within the article.
